# Metabolic Regulation of Invadopodia and Invasion by Acetyl-CoA Carboxylase 1 and *De novo* Lipogenesis

**DOI:** 10.1371/journal.pone.0029761

**Published:** 2012-01-06

**Authors:** Kristen E. N. Scott, Frances B. Wheeler, Amanda L. Davis, Michael J. Thomas, James M. Ntambi, Darren F. Seals, Steven J. Kridel

**Affiliations:** 1 Department of Cancer Biology, Wake Forest School of Medicine, Winston Salem, North Carolina, United States of America; 2 Department of Biochemistry, Wake Forest School of Medicine, Winston Salem, North Carolina, United States of America; 3 Comprehensive Cancer Center, Wake Forest School of Medicine, Winston Salem, North Carolina, United States of America; 4 Department of Biochemistry, University of Wisconsin-Madison, Madison, Wisconsin, United States of America; Laurentian University, Canada

## Abstract

Invadopodia are membrane protrusions that facilitate matrix degradation and cellular invasion. Although lipids have been implicated in several aspects of invadopodia formation, the contributions of *de novo* fatty acid synthesis and lipogenesis have not been defined. Inhibition of acetyl-CoA carboxylase 1 (ACC1), the committed step of fatty acid synthesis, reduced invadopodia formation in Src-transformed 3T3 (3T3-Src) cells, and also decreased the ability to degrade gelatin. Inhibition of fatty acid synthesis through AMP-activated kinase (AMPK) activation and ACC phosphorylation also decreased invadopodia incidence. The addition of exogenous 16∶0 and 18∶1 fatty acid, products of *de novo* fatty acid synthesis, restored invadopodia and gelatin degradation to cells with decreased ACC1 activity. Pharmacological inhibition of ACC also altered the phospholipid profile of 3T3-Src cells, with the majority of changes occurring in the phosphatidylcholine (PC) species. Exogenous supplementation with the most abundant PC species, 34∶1 PC, restored invadopodia incidence, the ability to degrade gelatin and the ability to invade through matrigel to cells deficient in ACC1 activity. On the other hand, 30∶0 PC did not restore invadopodia and 36∶2 PC only restored invadopodia incidence and gelatin degradation, but not cellular invasion through matrigel. Pharmacological inhibition of ACC also reduced the ability of MDA-MB-231 breast, Snb19 glioblastoma, and PC-3 prostate cancer cells to invade through matrigel. Invasion of PC-3 cells through matrigel was also restored by 34∶1 PC supplementation. Collectively, the data elucidate the novel metabolic regulation of invadopodia and the invasive process by *de novo* fatty acid synthesis and lipogenesis.

## Introduction

Podosomes and invadopodia are membrane protrusions at focalized sites of polymerized actin that coordinate the invasive properties or normal and tumor cells, respectively [1]. In addition to polymerized actin, both structures comprise a complex assortment of proteins including actin-modifying proteins, integrins, proteases, multiple kinases, and several scaffolding proteins [2]. The integration of these components facilitates the invasive process through coordinated proteolysis of the extracellular matrix [3]. Podosomes have been visualized on invading cells *ex vivo* and disruption of podosome architecture decreases the ability of cells to invade [1], [4]. Because of their complex makeup, both structures can be regulated through multiple mechanisms including, but not limited to, kinase signaling and reactive oxygen species [1], [5]. Given the complex architecture of podosomes and invadopodia it is likely that other processes underlying podosome and invadopodia construction remain to be elucidated.

The dynamic cell membrane is what connects the extracellular function of podosomes and invadopodia to their intracellular architecture. Several pieces of evidence support a role for lipids and cellular membranes in the formation of podosome and invadopodia. First, the phosphoinositide (PI) class of lipids is critical to the formation of podosomes and invadopodia [6], [7]. Specifically, invadopodia formation is initiated when PI(3,4)P_2_ accumulates at focal adhesions following Src activation [8]. Related to this, the p110α catalytic subunit of PI3-kinase is necessary for the formation of invadopodia in breast MDA-MB-231 cells [9]. Given this, it is no surprise that several podosome and invadopodia-associated proteins have lipid-binding domains. For example, tyrosine kinase substrate 5 (Tks5) has a phox homology (PX) domain that facilitates interactions with PI(3)-P and PI(3,4)-P_2_ [10]. Cholesterol is another lipid that has been implicated in invadopodia formation. Particularly, cholesterol levels regulate caveolin-1 interactions and lipid raft formation to provide an appropriate scaffold for invadopodia to form [11], [12]. Lastly, actin polymerization is dependent on appropriate membrane curvature and the function of membrane associated BAR (bin-amphiphysin-Rvs) domain proteins [13]. Collectively, this body of literature provides compelling evidence that lipid composition and membrane dynamics occupy a critical role in mediating the formation and activity of podosomes and invadopodia.

The *de novo* fatty acid synthesis pathway is an important component of the metabolic phenotype of tumor cells. Fatty acids are synthesized from acetyl-CoA and malonyl-CoA by fatty acid synthase (FASN) [14]. The rate limiting step of the pathway, however, is the production of malonyl-CoA by acetyl-CoA carboxylase 1 (ACC1) for both synthesis and elongation of fatty acids [15], [16]. Although the pathway provides fatty acid that can be used for multiple processes, the majority of *de novo* synthesized fatty acid is incorporated into phospholipids for membrane biogenesis [14], [17]. Thus, the fatty acid synthesis pathway is a significant contributor to the cellular membrane. A major fraction of newly synthesized fatty acids localize to lipid rafts, suggesting that they regulate crucial signaling and membrane-based processes [17]. As a result, the fatty acid synthesis pathway could provide a metabolic cue for membrane-mediated processes like podosome and invadopodia facilitated invasion.

A body of literature has demonstrated that the expression and activity of enzymes in the fatty acid synthesis pathway are significantly increased in most cancers [18]. Moreover, the expression levels of ACC1 and FASN have been correlated with aggressive disease and poor outcome [19], [20], [21], [22], [23], [24], [25], [26], [27], [28], [29], suggesting an important role for this pathway in advanced disease. Because invadopodia are membrane protrusions and aspects of lipids and lipid metabolism have been implicated in their function, we investigated the role of *de novo* fatty acid synthesis in invadopodia formation and function. Here we describe the novel connection between ACC1 activity, fatty acid synthesis, and invadopodia function in Src-transformed 3T3 cells, specifically, through modulation of fatty acid and phospholipid synthesis. These results provide a novel mechanism by which *de novo* lipogenesis regulates the invasive process of tumor cells.

## Materials and Methods

### Materials

5-(Tetradecyloxy)-2-furoic acid (TOFA) and 5-Aminoimidazole-4-carboxamide ribonucleotide (AICAR) was purchased from Calbiochem (La Jolla, CA). CP-640186 was a generous gift from Pfizer (Groton, CT). Fatty acid free bovine serum albumin (BSA), 16∶0 fatty acid sodium salt, BSA-18∶1 fatty acid, and all standard chemicals were purchased from Sigma-Aldrich (St. Louis, MO). 1-myristoyl-2-palmitoyl-*sn*-glycero-3-phosphocholine (30∶0 PC), 1-palmitoyl-2-oleoyl-*sn*-glycero-3-phosphocholine (34∶1 PC), and 1,2-dioleoyl-*sn*-glycero-3-phosphocholine (36∶2 PC) were purchased from Avanti Polar Lipids (Alabaster, AL). Antibodies against acetyl-CoA carboxylase1, p-ACC1S79, AMP-activated kinase (AMPK), p-AMPKT172, cortactin, p-SrcY416, and Src were purchased from Cell Signaling Technologies (Beverly, MA). Antibody against fatty acid synthase was purchased from BD Transduction Labs (San Diego, CA). Antibody against β-actin was purchased from Sigma-Aldrich (St. Louis, MO). Antibody against neural Wiskott-Aldrich syndrome protein (N-WASp) was purchased from Santa Cruz Biotechnology (Santa Cruz, CA). Secondary antibodies were purchased from Bio-Rad Laboratories (Hercules, CA). Cell culture and AlexaFluor reagents were purchased from Invitrogen (Carlsbad, CA).

### Cell Lines and Culture Conditions

The established cell lines MDA-MB-231, PC-3, Snb19 and 293T were obtained from the American Type Tissue Collection (ATCC). The human cell lines for this study were determined to be exempt by the Institutional Review Board of Wake Forest University. Wild type and stearoyl-CoA desaturase-1 deficient cell lines (SCD-1^+/+^ and SCD-1^−/−^) were described previously [30]. Parental 3T3 and 3T3+Src(Y527F) were described previously [10]. The 3T3, 3T3+Src(Y527F), MDA-MB-231, and Snb19 cells were maintained in DMEM. The PC-3 cells were maintained in RPMI-1640. All media were supplemented with 10% fetal bovine serum (FBS), penicillin/streptomycin, and cultured in 5% CO_2_ at 37°C. Primary SCD-1^+/+^ and SCD-1^−/−^ MEFs were immortalized using the 3T3 growth crisis protocol [31]. The pBABE/puro-Src(Y527F) retroviral vector was transfected into 293T packaging cells with lipofectamine 2000 (Invitrogen, Carlsbad, CA). Conditioned media containing viral particles was harvested then applied to SCD-1^+/+^ and SCD-1^−/−^ MEFs in a 1∶1 ratio in medium supplemented with 4 µg/mL polybrene. The MEFs were infected for 24 hours then selected with 2 µg/mL puromycin in normal growth medium followed by maintenance in growth medium containing 1 µg/mL puromycin. Levels of p-Src(Y416) and total Src were analyzed by immunoblot (see *Immunoblot Analysis*) in stable populations of SCD1^+/+^-Src and SCD1^−/−^-Src cells.

### Metabolic Labeling

3T3 and 3T3-Src cells were seeded in 24-well plates at a density of 8×10^4^ cells/well. After 24 hours the cells were treated with vehicle (Dimethyl sulfoxide (DMSO, 0.1%) or TOFA (30 µM) for 2 hours. In the AMPK studies, the cells were treated with vehicle (dH_2_O) or AICAR (2.5 mM) for 16 hours. To label newly synthesized fatty acid, cells are pulsed with 1 µCi ^14^C-acetate (GE Healthcare, Piscataway, NJ) for 2 hours, washed with phosphate buffered saline (PBS), and lysed in hypotonic buffer (1 mM dithiothreitol (DTT), 1 mM ethylenediaminetetraacetic acid (EDTA), 20 mM Tris, pH 7.5). Lipids were extracted with chloroform∶methanol (2∶1 v/v) and ^14^C-acetate incorporation was quantified by scintillation counting as previously described [32].

### Invadopodia Visualization

For inhibitor studies, 3T3-Src cells were seeded in 6-well plates containing glass cover slips at a density of 1×10^5^ cells/well then treated with vehicle (DMSO 0.1%), TOFA (30 µM), or CP640186 (100 µM) for 24 hours before coverslip collection. For siRNA studies, ACC was knocked down as outlined in the following section for 48 hours before coverslip collection. For AMPK studies, the pre-plated cells were treated with AICAR (2.5 mM) for 16 hours. For fatty acid rescue experiments, 3T3-Src cells were treated with vehicle (DMSO 0.1%, 1% BSA) and TOFA, or AICAR in the absence or presence of 300 µM 16∶0 or 18∶1 fatty acid. For the lipid rescue experiments, cells were treated with vehicle (DMSO 0.1%-EtOH 0.04%) and TOFA or AICAR, in the absence or presence of 150 µM 30∶0 PC or 300 µM 34∶1 or 36∶2 PC. For the experiments examining the role of SCD-1 in invadopodia, SCD1^+/+^-Src and SCD1^−/−^-Src cells were seeded at 1×10^5^ cells/well and grown on glass coverslips for 24 hours before coverslip collection. The coverslips were washed with PBS and fixed with 4% paraformaldehyde. The cells were permeablized with 0.1% Triton X-100 in PBS followed by staining with AlexaFluor488-phalloidin in 5% donkey serum-PBS for 1 hour and 4′,6-diamidino-2-phenylindole (DAPI, 1 µg/mL in PBS for 5 min) before mounting onto glass slides. For co-staining, fixed cells were incubated with a primary antibody against cortactin in 5% donkey serum-PBS for 1 hour followed by incubation with AlexaFluor488-phalloidin, 5% donkey serum-PBS for 1 hour, AlexaFluor594-secondary antibody in 5% donkey serum-PBS for 1 hour, and DAPI (1 µg/mL in PBS for 5 min). The coverslips were mounted onto glass slides. Images were collected in the Cell Analysis core laboratory using a Zeiss Axioplan 2 fluorescent microscope equipped with a 40× Plan-Neofluor (numerical aperture 1.3) and 63× PlanApochromat (numerical aperture 1.4) objectives, a Zeiss AxioCam digital camera, and Axioplan 2.05 imaging software. All merging and linear processing of images was done using Adobe Photoshop CS2. Invadopodia were defined as punctuate dots and rosettes were defined as O-like structures and quantified by counting a minimum of 200 cells per treatment.

### siRNA Knockdown of ACC

3T3-Src cells were plated into 10 cm dishes. After 24 hours, the cells were transfected with 100 nM of either of two independent siRNAs specific for ACC1 (siRNA#1, GUAGAAAUCAAAUUCCGUAUUUU or siRNA#2, CAAGACUGAUGGCGAUAUU) (Dharmacon, Lafayette, CO) or scrambled siRNA (100 nM) using either siPORT NeoFX (Ambion, Austin, TX) or Lipofectamine 2000 (Invitrogen, Carlsbad, CA). After 48 or 72 hours, the cells were processed for invadopodia visualization or invasion assay, respectively, and protein was harvested for analysis by immunoblot (see *Immunoblot Analysis*). In the case of fatty acid and lipid supplementation, the indicated fatty acid or lipid were added 24 hours before processing the cover slips.

### Immunoblot Analysis

Protein was harvested by scraping cells into media then pelleting by centrifugation. The cells were washed with ice cold PBS then lysed in ice cold buffer (20 mM Tris, pH 8.3, 5 mM EDTA, 1% Triton X-100) supplemented with a protease and phosphatase inhibitor cocktail (5 µg/mL aprotinin, 5 µg/mL leupeptin, 5 µg/mL pepstatin A, 1 mM sodium orthovanadate, 1 mM sodium fluoride, 0.1 µM okadeic acid, and 200 µM phenylmethylsulfonyl fluoride). Proteins were separated by SDS-PAGE through 13.5%, 10%, or 7.5% gels and transferred to nitrocellulose membrane. After incubation with appropriate antibodies, immunoreactive proteins were detected with enhanced chemiluminescence (Perkin Elmer, Waltham, MA). β-actin or β-tubulin was used as loading control. Immunoreactive bands were quantified by densitometry using the ImageJ program.

### Phospholipid Analysis

3T3-Src or PC-3 cells were treated with vehicle (DMSO 0.1%) or TOFA (30 µM) for 24 hours then the treated cells were trypsinized, pelleted by centrifugation, washed with ice cold 0.9% saline, and snap frozen in liquid nitrogen. Lipid was extracted from the cell pellets using the method of Bligh and Dyer [33] and suspended in 1∶1 chloroform∶methanol (v∶v). The total phosphorous levels were determined according to the method of Rouser *et al* [34]. The lipid samples were diluted to 2 nmol/mL phosphorous with chloroform/methanol containing 1% formic acid. The phospholipid profiles were analyzed using a TSQ Discovery Max electrospray ionization triple quadrupole mass spectrometer using the specifications outlined by DeLong, *et al* [35]. The PC, PE and PS profiles were analyzed in the positive ion mode by analyzing the spectra around the neutral loss of *m/z* 184.1, 141.1, and 185.1, respectively. The PI profile was analyzed in the negative ion mode by analyzing the precursor spectra of *m/z* 241.1.

Phospholipid classes were analyzed as outlined by Van Kessel, *et al.* [36] in 3T3-Src or PC-3 cells treated with vehicle (DMSO 0.1%) or TOFA (30 µM) for 24 hours. Briefly, cellular lipids were extracted as above then applied to a 300 mm Waters μPorasil column that has a 10 µm particle size. Lipid classes were separated using gradient elution with the following buffers: A - hexane : isopropanol : water : ammonium hydroxide (150∶200∶5∶2, v/v); B - hexane : isopropanol : water : ammonium hydroxide (150∶200∶35∶2, v/v); and C - chloroform : isopropanol (1∶1 v/v). Total phosphorus content of each lipid class was determined according to Rouser, *et. al* [34]. The absolute pmol phospholipid in each sample was calculated from the mol% reading and the phospholipid class analysis then normalized to nmol phosphorus (P).

### Invasion Assays

3T3-Src (2×10^4^), MDA-MB-231 (2×10^4^), Snb19 (2×10^4^), or PC-3 (1.75×10^4^) cells were seeded into the top well of an invasion chamber fitted with a matrigel coated porous membrane (BD Biocoat, Franklin Lakes, NJ) in media with 0.1% FBS. For inhibitor studies, the cells were seeded in the presence of vehicle (DMSO 0.1%) or TOFA (30 µM). For lipid rescue studies, the cells were seeded in the presence of the following: vehicle (DMSO 0.1%, EtOH 0.04%); TOFA (30 µM), or TOFA plus 300 µM 34∶1 or 36∶2 PC species. Invasion assays were incubated for 24 hours. Cells that migrated to the underside of the membrane were fixed with 10% methanol∶10% acetic acid (v∶v) for 30 min, stained with 0.4% crystal violet (30 min) and counted.

### Gelatin Degradation and Zymography Analysis

3T3-Src cells were plated in 6-well plates with glass coverslips coated with AlexaFluor488 labeled gelatin at a density of 1.5×10^5^ cells/well [37]. The cells were treated with vehicle (0.1% DMSO) or TOFA (30 µM) and gelatin degradation proceeded for 24 hours. The cells were then fixed with paraformaldehyde and stained with AlexaFluor594-phalloidin and DAPI as outlined in *Invadopodia Visualization*. For siRNA experiments, the cells were treated with ACC1-targeting siRNA for 24 hours (as in *siRNA knockdown of ACC1*) then plated onto gelatin-coated coverslips and processed as above. For AMPK studies, 3T3-Src cells were treated with AICAR (2.5 mM) for 16 hours then processed as above. Fatty acid and lipid rescue experiments were performed as described above. Degradation area per cell was determined using ImageJ. For zymography analysis, 3T3-Src cells were seeded in 6-well plates at a density of 2.0×10^5^ cells per well. After 6 hours the media was replaced with OptiMem reduced serum media. After 16 hours, the cells were treated with vehicle (DMSO 0.1%, 1% BSA), TOFA (30 µM), or TOFA +75, 150, or 300 µM 18∶1 fatty acid. After 48 hours the conditioned media was removed and proteins were resolved a 10% zymography gel. The gel was incubated in PBS buffer containing 2.5% Triton X-100 then developed for 24 hours at 37°C in buffer containing 50 mM Tris, pH 7.5, 5 mM CaCl_2_·2H_2_O, 200 mM NaCl, 0.02% Brij-35. The zymogram was stained in buffer containing 40% methanol, 10% acetic acid, and 0.5% Coomassie Blue R-250 followed by destaining in buffer containing 40% methanol and 10% acetic acid in dH_2_O.

### Clonogenic and Proliferation Studies

3T3-Src cells were plated into 6-well plates at 400 cells/well in the presence of vehicle (DMSO 0.1%) or TOFA (30 µM). The cells were treated for 16 hours, then the media was replaced with normal growth medium and colonies developed for 6–9 days. The colonies were fixed with 10% methanol and 10% acetic acid for 10 min followed by staining with 0.4% crystal violet for 30 min, washed, dried and quantified by counting. To measure proliferation 3T3-Src cells were seeded at a density of 5×10^4^ cells/well and allowed to adhere for 24 hours. The cells were treated with vehicle (DMSO 0.1%) and TOFA (30 µM) for 24 hours. For counting, the cells were trypsinized, pelleted by centrifugation and resuspended in cold PBS/trypan blue. Viable cells were determined by exclusion of trypan blue.

### Statistical Analysis

Statistical significance between two groups was determined by Student's t-test. Error bars for all graphs represent standard deviation except the graphs quantifying gelatin degradation, which represents standard error of the mean.

## Results

### Acetyl-CoA carboxylase 1 activity is required for invadopodia

**Figure 1:**
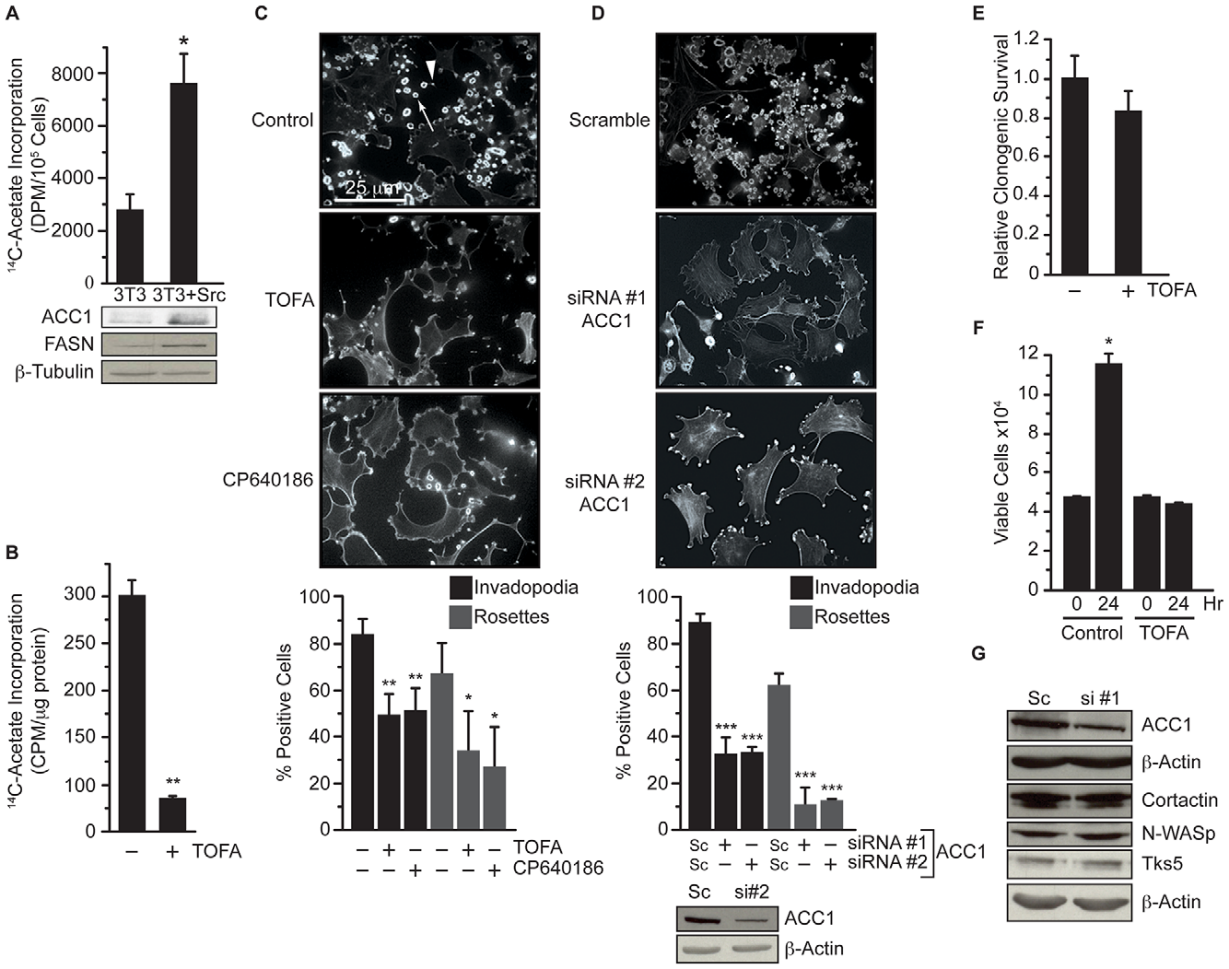
ACC1 activity is required for invadopodia formation. (A) Fatty acid synthesis was determined by incorporation of ^14^C-acetate into lipid in 3T3 and 3T3-Src cells. Protein expression of ACC1, FASN, and β-tubulin were determined by immuno-blot. (B) 3T3-Src cells were treated with vehicle or TOFA (30 µM) for 2 hours and fatty acid synthesis was determined as in A. (C) 3T3-Src cells were treated with vehicle, TOFA (30 µM), or CP640186 (100 µM) for 24 hours. Invadopodia and rosettes were visualized by staining cells with AlexaFluor488-phalloidin. Arrowhead indicates punctate invadopodia and arrow indicates rosette superstructure. (D) 3T3-Src cells were transfected with 100 nM scrambled (Sc) or two independent ACC1-specific siRNAs (siRNA #1 or #2) for 48 hours. Invadopodia were quantified after AlexaFluor488-phalloidin staining. ACC1 and β-actin protein levels were analyzed by immune-blot. (E) 3T3-Src cells were treated with vehicle or TOFA (30 µM) for 16 hours and clonogenic survival was determined. (F) 3T3-Src cells were treated with vehicle or TOFA (30 µM) for 24 hours and viable cells were quantified by trypan blue exclusion. (G) 3T3-Src cells were transfected with 100 nM scrambled or siRNA #1 against ACC1 as in D. Expression of the indicated proteins was analyzed by immunoblot. Scale bar, 25 µm. *p≤0.05; **p≤0.01; ***p≤0.001.

To determine whether fatty acid synthesis has a role in Src mediated invadopodia formation, the expression and activity of the fatty acid synthesis pathway was determined in 3T3 cells and 3T3 cells transformed with constitutively active Src (3T3-Src) [38]. Transformation with Src increased fatty acid synthesis approximately three-fold and also increased ACC1 and FASN protein levels (Fig. 1A, p≤0.05). Inhibition of ACC in 3T3-Src cells by the non-isozyme selective inhibitor 5-(Tetradecyloxy)-2-furoic acid (TOFA) decreased fatty acid synthesis by approximately 83% (Fig. 1B, p≤0.01). Pharmacological inhibition of ACC activity with TOFA or CP-640186 [39] resulted in a dramatic reduction in invadopodia incidence (Fig. 1C). TOFA treatment reduced punctate invadopodia (indicated by arrowhead in control micrograph) by 41% and rosettes (indicated by arrow in control micrograph) by 49% (p≤0.05), and CP-640186 treatment reduced invadopodia by 39% and rosettes by 60% (p≤0.05). Along with invadopodia loss, pharmacological inhibition of ACC also induced morphological changes such as cell enlargement (Fig. 1C). Because TOFA and CP-640186 inhibit both ACC1 and ACC2, we also assessed invadopodia following specific ACC1 knockdown with siRNA because of its role in fatty acid synthesis. Transfection of 3T3-Src cells with two independent siRNAs against ACC1 reduced invadopodia incidence by 63.5% and rosettes by 83% (p≤0.0005) compared to cells transfected with scrambled siRNA (Fig. 1D). Although TOFA treatment did not have a significant impact on clonogenic survival, it did reduce proliferation of the Src transformed cells (Fig. 1E&F). Interestingly, knockdown of ACC1 reduced invadopodia incidence, independent of changes in expression of invadopodia-associated proteins like cortactin, N-WASp and Tks5 (Fig. 1G). Together, these data demonstrate that ACC1 is required for proper invadopodia assembly.

### Restoration of invadopodia incidence by fatty acid

**Figure 2:**
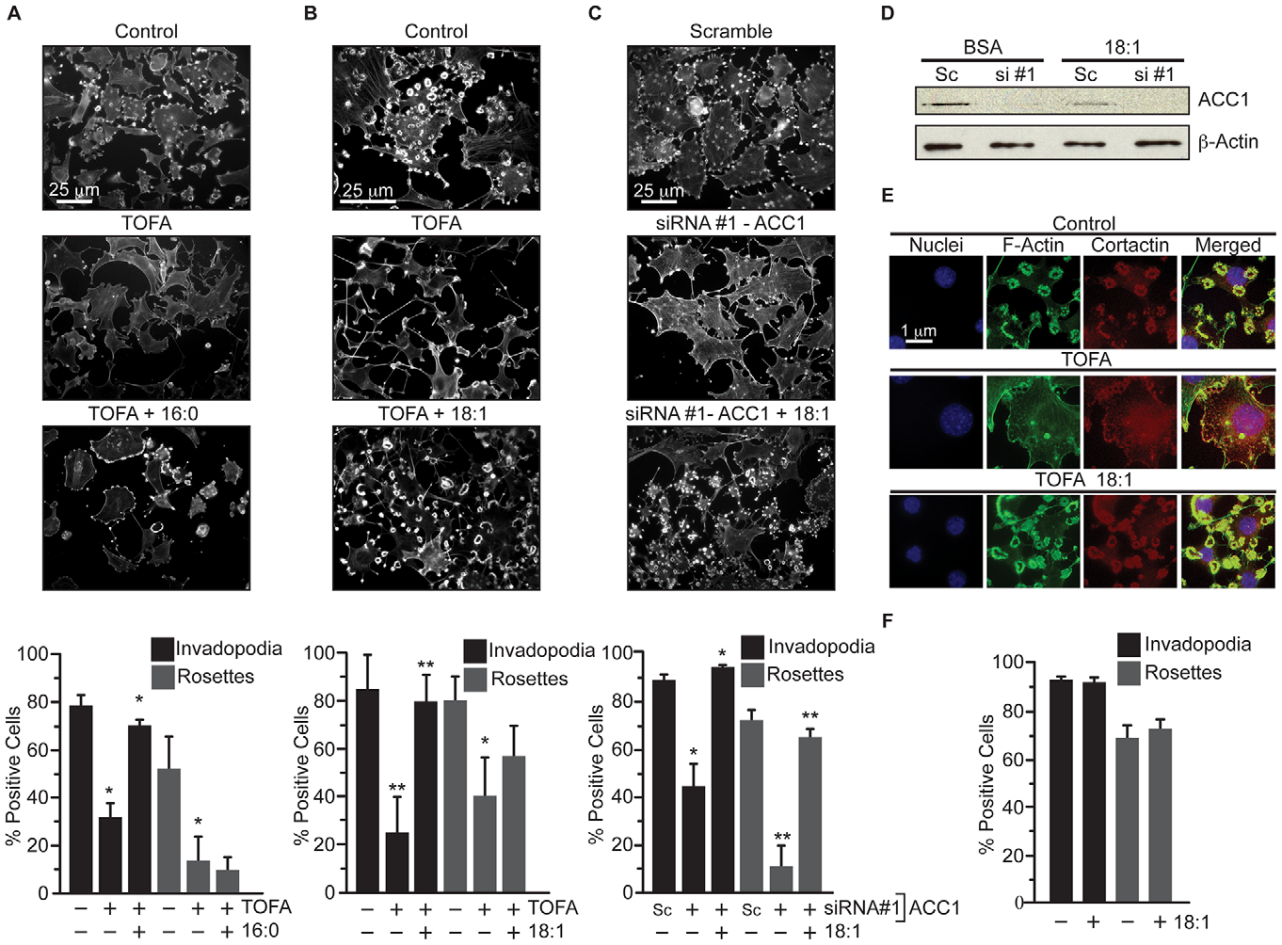
Fatty acid restores invadopodia following ACC1 inhibition. (A) Invadopodia incidence in 3T3-Src cells treated with vehicle, TOFA (30 µM), or TOFA +16∶0 fatty acid for 24 hours. (B) Invadopodia incidence in 3T3-Src cells treated with vehicle, TOFA (30 µM), or TOFA +18∶1 fatty acid for 24 hours. (C) Invadopodia incidence in 3T3-Src cells transfected with 100 nM scrambled (Sc) siRNA or siRNA #1 against ACC1 for 48 hours and then supplemented with 18∶1 fatty acid for 24 hours. (D) ACC1 protein expression in 3T3-Src cells transfected with 100 nM scrambled or siRNA #1 against ACC1 and supplemented with 18∶1 fatty acid. (E) Cortactin localization to invadopodia in 3T3-Src cells treated with vehicle, TOFA (30 µM), or TOFA supplemented with 18∶1 fatty acid for 24 hours. Cortactin co-localization with F-actin was determined by immunofluorescence. Scale bar,1 µm. (F) 3T3-Src cells were treated with BSA or 18∶1 fatty acid for 24 hours and invadopodia incidence was determined. Scale bar, 25 µm *p≤0.05; **p≤0.01.

Because ACC1 catalyzes the rate-limiting step of fatty acid synthesis (Fig. 1B), the ability of exogenous fatty acid to restore invadopodia incidence in TOFA-treated 3T3-Src cells was determined. The addition of 16∶0 fatty acid (palmitate) increased the percentage of TOFA-treated cells making invadopodia from 40% of control to 89% of control (p≤0.02, Fig. 2A). Palmitate did not restore rosette formation, however (Fig. 2A). Supplementation with 18∶1 fatty acid (oleate) increased the percentage of cells with invadopodia from 29.6% of control to 95.0% of control and the percentage of cells with rosettes to 72% of control (p≤0.01, Fig. 2B). The ability of 18∶1 fatty acid to restore invadopodia in cells transfected with two ACC1-specific siRNAs was also determined. The addition of 18∶1 fatty acid to cells transfected with siRNA against ACC1 completely restored the incidence of cells making invadopodia and rosettes (Fig. 2C, p≤0.02), without affecting ACC1 knockdown (Fig. 2D). It is worth noting that the addition of 18∶1 fatty acid to 3T3-Src cells did not affect invadopodia formation on its own (Fig. 2F). The addition of 18∶1 fatty acid to TOFA-treated cells also restored cortactin localization to focalized actin in invadopodia (Fig. 2E). These data support the notion that *de novo* fatty acid synthesis is required for maintenance of invadopodia structures in 3T3-Src cells.

**Figure 3:**
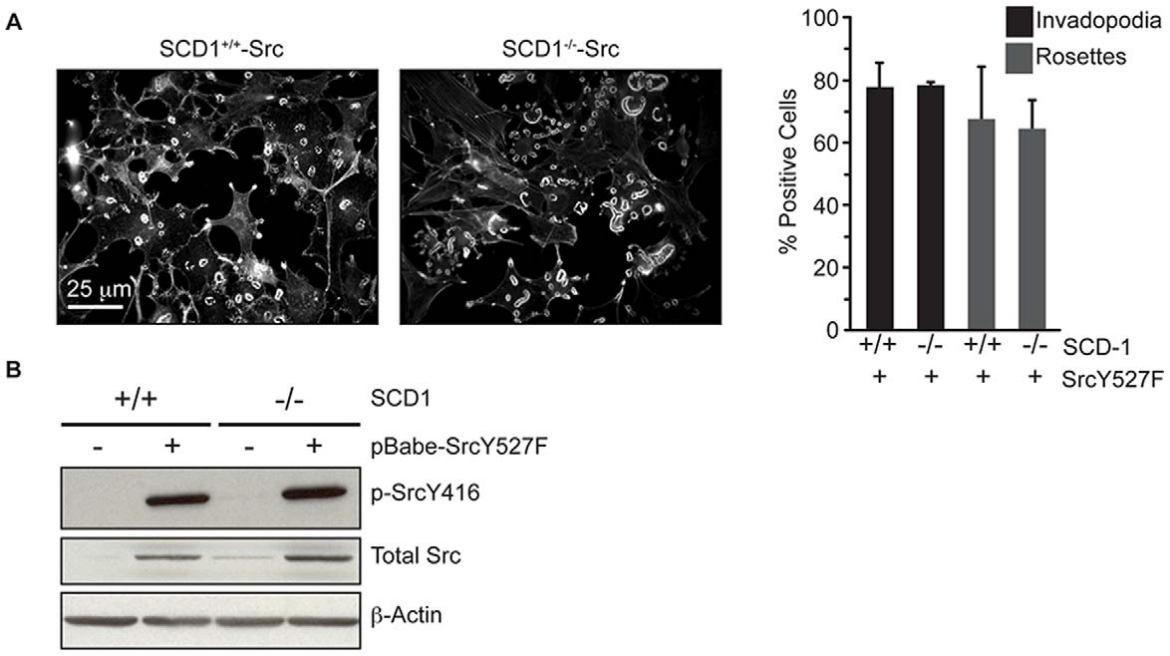
Stearoyl-CoA desaturase-1 is not required for invadopodia formation. (A) Invadopodia incidence was determined in wild-type (SCD1^+/+^) and SCD-1 deficient (SCD1^−/−^) MEFs transformed with Src(Y527F). (B) SCD1^+/+^ and SCD1^−/−^ MEFs transformed with Src(Y527F) were harvested, lysed, and p-SrcY416, total Src and β-actin levels were determined by immunoblot blot. Scale bar, 25 µm.

Because 18∶1 fatty acid is able to restore both invadopodia and rosettes in cells with disrupted ACC1 activity, we determined whether loss of stearoyl-CoA desaturase 1 (SCD-1), the enzyme that generates 18∶1, affects invadopodia incidence. Wild-type and SCD-1deficient MEFs [30] were transformed with constitutively active Src (Src-SCD-1^+/+^ and Src-SCD-1^−/−^) and invadopodia incidence was quantified. The absence of SCD-1 had no effect on invadopodia incidence (Fig. 3A, p≤0.90), Src expression or SrcY416 phosphorylation (Fig. 3B). These data imply that the effects of ACC1 inhibition on invadopodia are not solely due to loss of 18∶1 levels, but rather to decreases in total fatty acid synthesis.

### Inhibition of ACC1 affects the lipid profile of 3T3-Src cells

To elucidate the connection between lipogenesis and invadopodia the lipid profile of 3T3-Src cells treated with TOFA was determined by mass spectrometry. Blockade of ACC activity most dramatically altered the phosphatidylcholine (PC) profile of 3T3-Src cells. Consistent with the role of ACC1 in *de novo* fatty acid synthesis, TOFA treatment reduced the levels of virtually all PC species containing saturated and monounsaturated fatty acids (Table 1). For example, 30∶0 PC levels decreased from 19.7 pmol/nmol phosphorous to 6.6 pmol/nmol phosphorous, 34∶1 PC decreased from 78.2 pmol/nmol phosphorous to 47.6 pmol/nmol phosphorous and 36∶2 PC decreased from 28.6 pmol/nmol phosphorous to 18.3 pmol/nmol phosphorous. Conversely, inhibition of ACC activity resulted in a shift away from the saturated and monounsaturated species towards the polyunsaturated species as has been demonstrated previously [40]. Among the increased polyunsaturated species, the largest increases were observed in the 36∶4, 36∶5, and 38∶5 PC species with each increasing approximately 2-fold (Table 1, p<0.0001). Taken together, these data demonstrate that ACC activity is important for maintaining the PC composition in 3T3-Src cells.

**Table 1:** ACC inhibition alters the phospholipid profiles of 3T3-Src cells.

	PC (pmol/nmol P)		PE (pmol/nmol P)		PS (pmol/nmol P)		PI (pmol/nmol P)	
	Control	TOFA	Control	TOFA	Control	TOFA	Control	TOFA
**30∶0**	19.7±0.8	6.6±0.2*	-	-	-	-	-	-
**30∶1**	6.9±0.4	2.3±0.5*	-	-	-	-	-	-
**32∶0**	31.4±1.3	25.6±0.3*	-	-	-	-	-	-
**32∶1**	64.5±1.6	24.4±0.7*	-	-	0.3±0.2	0.6±0.5	-	-
**32∶2**	11.3±0.5	3.8±0.2*	-	-	-	-	-	-
**32∶3**	-	-	-	-	1.4±0.5	0.3±0.4	-	-
**32∶4**	-	-	-	-	1.1±0.4	1.0±0.6	-	-
**34∶0**	7.8±0.4	8.6±0.5*	0.8±0.6	0.5±0.6	-	-	-	-
**34∶1**	78.2±2.2	47.6±1.1*	9.9±2.5	3.0±1.0*	3.5±2.4	2.2±0.5	-	-
**34∶2**	40.3±1.2	20.3±0.6*	5.8±1	0.8±0.5*	0.7±0.5	0.6±0.4	-	-
**34∶3**	5.0±0.05	3.8±0.3*	-	-	1.8±0.8	0.9±0.7	-	-
**34∶4**	-	-	-	-	5.9±1.1	1.8±0.9*	-	-
**34∶5**	-	-	-	-	0.8±0.4	0.3±0.3	-	-
**36∶0**	2.1±1.4	2.8±0.4	-	-	-	-	-	-
**36∶1**	9.9±0.8	8.1±0.4*	-	-	5.9±1.1	3.2±0.9	0.7±0.8	0.4±0.8
**36∶2**	28.6±0.9	18.3±0.8*	0.8±0.6	0.5±0.6	2.1±0.7	1.4±0.5*	5.5±1.9	1.9±1.0*
**36∶3**	9.2±0.3	17.8±0.5*	9.9±2.5	3.0±1.0*	1.5±1.0	0.8±0.6	3.4±2.3	0.6±0.8
**36∶4**	7.9±0.3	36.6±1.9*	5.8±1	0.8±0.5*	4.4±1.5	1.8±0.8	2.3±0.6	0.8±0.5*
**36∶5**	2.3±0.2	9.1±0.2*	-	-	2.2±1.7	1.5±0.4*	-	-
**38∶0**	3.6±0.2	3.3±0.3	-	-	-	-	-	-
**38∶1**	-	-	-	-	-	-	0.6±0.9	0.3±0.6
**38∶2**	1.1±1.3	2.8±0.6	2.1±0.8	1.1±0.2*	-	-	-	-
**38∶3**	4.0±0.8	7.4±0.8*	8.1±3.0	5.8±0.7	1.3±0.7	1.9±0.8*	8.9±3.2	8.9±2.7
**38∶4**	9.4±0.4	30.9±0.9*	33.5±3.6	35.8±0.7	1.7±0.6	2.5±0.7*	35.3±9.7	37.1±4.7
**38∶5**	9.9±0.5	42.0±1.2*	15.4±2.6	18.7±2.4	2.4±1.1	1.5±0.6	15.3±4.0	11.0±3.7
**38∶6**	4.0±0.2	10.9±0.5*	3.2±1.3	5.9±1.7*	0.6±0.6	1.7±0.6	-	-
**38∶7**	-	-	1.2±0.6	2.3±0.8	0.7±0.8	3.6±2.0	-	-
**38∶8**	-	-	0.8±0.6	1.3±0.3	-	-	-	-
**40∶4**	-	-	4.2±1.4	3.8±0.5	2.0±0.8	1.7±0.8	-	-
**40∶5**	2.9±0.3	6.4±0.3*	6.7±1.7	9.9±1.5*	4.0±1.6	5.1±0.7	1.2±1.4	5.6±1.6*
**40∶6**	4.1±0.2	8.1±0.2*	20.1±5.8	10.5±1.2*	4.0±1.2	3.2±2.1	1.1±0.8	5.0±1.0*
**40∶7**	3.2±0.3	6.4±0.5*	11.1±2.0	10.3±0.8	1.5±0.9	2.1±0.6	-	-
**40∶8**	-	-	1.8±0.7	4.3±0.8*	0.7±0.6	3.8±1.1	-	-
**40∶10**	-	-	-	-	0.3±0.3	0.6±0.2	-	-
**42∶9**	-	-	-	-	0.4±0.5	0.6±0.5	-	-
**42∶10**	-	-	-	-	0.5±0.6	0.8±0.3	-	-

- Not Detected.

*p≤0.05.

The profiles of phosphatidylethanolamine (PE), phosphatidylserine (PS), and phosphatidylinositol (PI) are also affected by ACC inhibition (Table 1). Interestingly, most of the species in these phospholipid classes did not contain saturated or mono-unsaturated fatty acids that would be derived from ACC1 activity. For example, the most abundant PE species, 38∶4 PE and 38∶5 PE, are not significantly changed following TOFA treatment. Similarly, the most abundant species of PI, 38∶4 PI, was not changed. The few species in each class that did contain saturated and mono-unsaturated fatty acid were reduced, and the species containing poly-unsaturated fatty acid were increased in relative abundance (Table 1). Overall, the effect of ACC inhibition in 3T3-Src on the PC profile suggests a role for PC in invadopodia formation.

**Figure 4:**
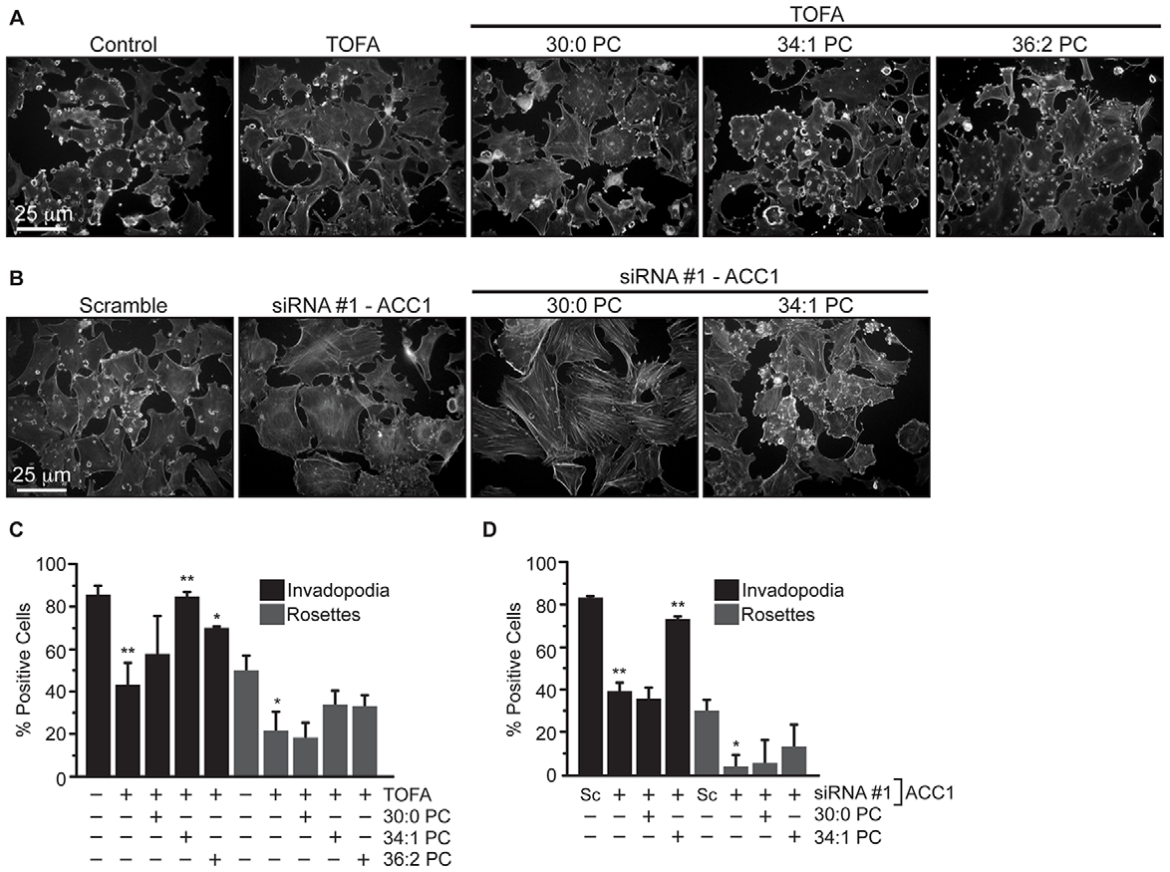
Specific phosphatidylcholine species restore invadopodia formation in 3T3-Src cells with impaired ACC activity. (A) Invadopodia incidence in 3T3-Src cells that were treated with vehicle, TOFA (30 µM), or TOFA supplemented 30∶0 PC, 34∶1 PC, or 36∶2 PC. (B) Invadopodia incidence in 3T3-Src cells transfected with 100 nM scrambled (Sc) or siRNA #1 against ACC1 and supplemented with 30∶0 PC or 34∶1 PC. (C) Quantification of A. (D) Quantification of B. Scale bar, 25 µm. *p≤0.05; **p≤0.01.

The contribution of specific PC species to invadopodia formation in cells with depleted ACC1 activity was also determined. We specifically focused on PC species because they are the most abundant phospholipid species, are a primary sink for *de novo* synthesized fatty acids, and were highly affected by ACC inhibition (Table 1) [17], [41]. Three PC species that represent small, moderate, and large percentages of total PC were used to determine the contribution of individual lipids to invadopodia formation. The ability of 30∶0 PC, 36∶2 PC, and 34∶1 PC to rescue invadopodia incidence following treatment with TOFA or transfection with ACC1-siRNA was determined. Although TOFA treatment reduced 30∶0 PC levels by 66% (Table 1), exogenous 30∶0 PC did not restore invadopodia in TOFA-treated 3T3-Src cells (Fig. 4A&C, p≤0.6), and higher concentrations of 30∶0 PC were toxic (data not shown). On the other hand, 34∶1 PC restored invadopodia incidence in TOFA-treated 3T3-Src cells from 59% to 99% of control (Fig. 4A&C, p≤0.002), and 36∶2 PC restored invadopodia incidence from 44% to 89% of control, respectively (Fig. 4A&C, p≤0.03). Interestingly, none of the PC species were able to completely rescue rosette superstructure formation although partial restoration was observed following exogenous 34∶1 and 36∶2 PC treatments (Fig. 4A&C). Similar results were obtained when these lipids were added to cells transfected with ACC1-specific siRNA (Fig. 4B&C). As in TOFA treated cells, the addition of 30∶0 PC to cells transfected with ACC1-specific siRNA did not restore invadopodia incidence (Fig. 4B&D). The addition of 34∶1 PC to 3T3-Src cells transfected with ACC1-directed siRNA restored invadopodia incidence to 88% of control levels (Fig. 4B&D). Rosette structures were not restored however (Fig. 4B&D). These data support a novel role for PC species in invadopodia assembly.

### ACC1 is required for invadopodia-mediated gelatin degradation

**Figure 5:**
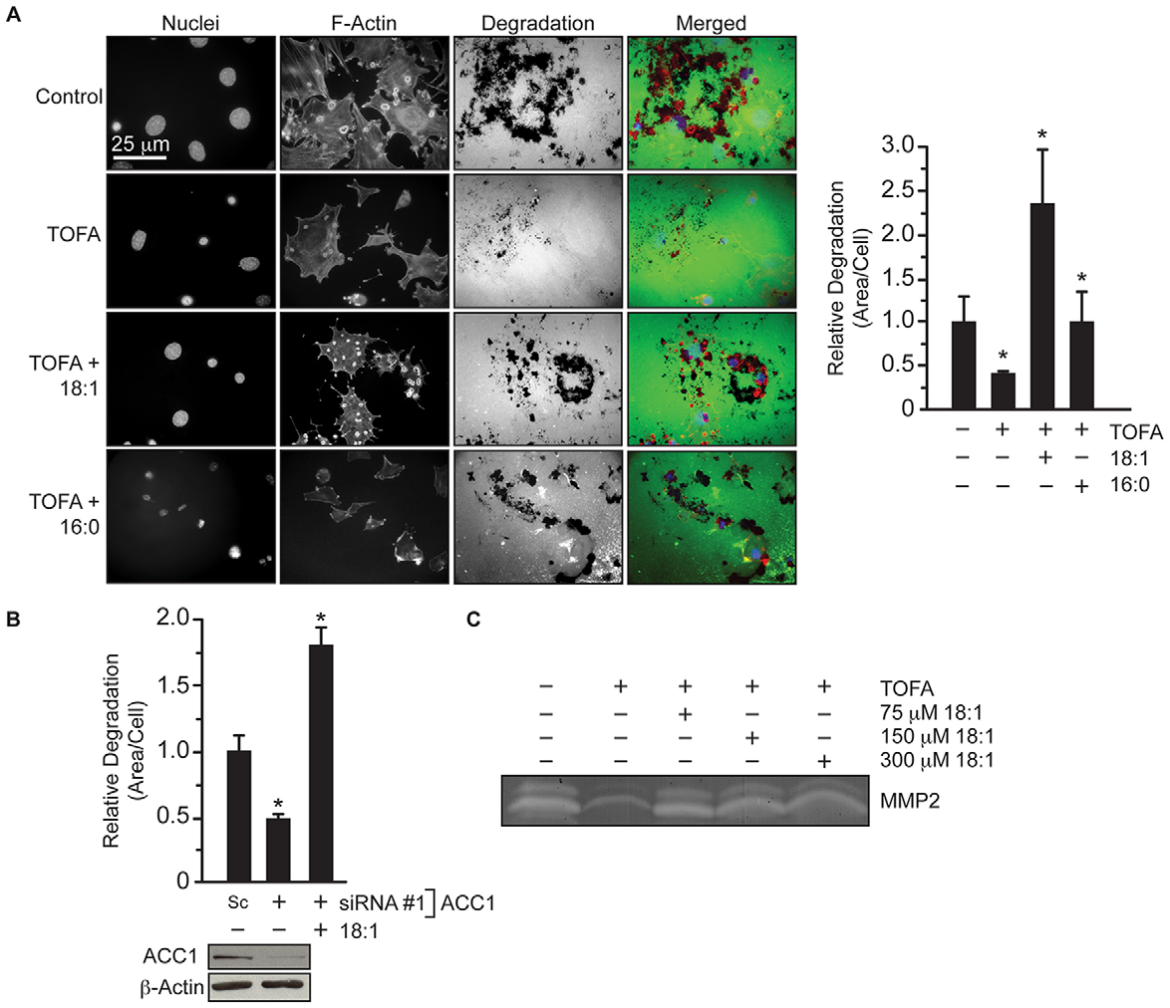
Fatty acid addition rescues gelatin degradation in 3T3-Src cells treated with TOFA. (A) Gelatin degradation of 3T3-Src cells seeded on glass cover slips coated with AlexaFluor-488 conjugated gelatin in the presence of vehicle, TOFA (30 µM), or TOFA with 18∶1 or 16∶0 fatty acid. Nuclei were visualized by staining with DAPI and actin was visualized with Alexafluor594-phalloidin. (B) Quantification of gelatin degradation by 3T3-Src cells transfected with scrambled (Sc) or siRNA #1 against ACC1 and supplemented with 18∶1 fatty acid for 24 hours. (C) Gelatin zymography of secreted MMP-2 in condition media from 3T3-Src cells treated with vehicle, TOFA (30 µM), or TOFA supplemented with 18∶1 fatty acid for 48 hours. Scale bar, 25 µm. *p≤0.05.

**Figure 6:**
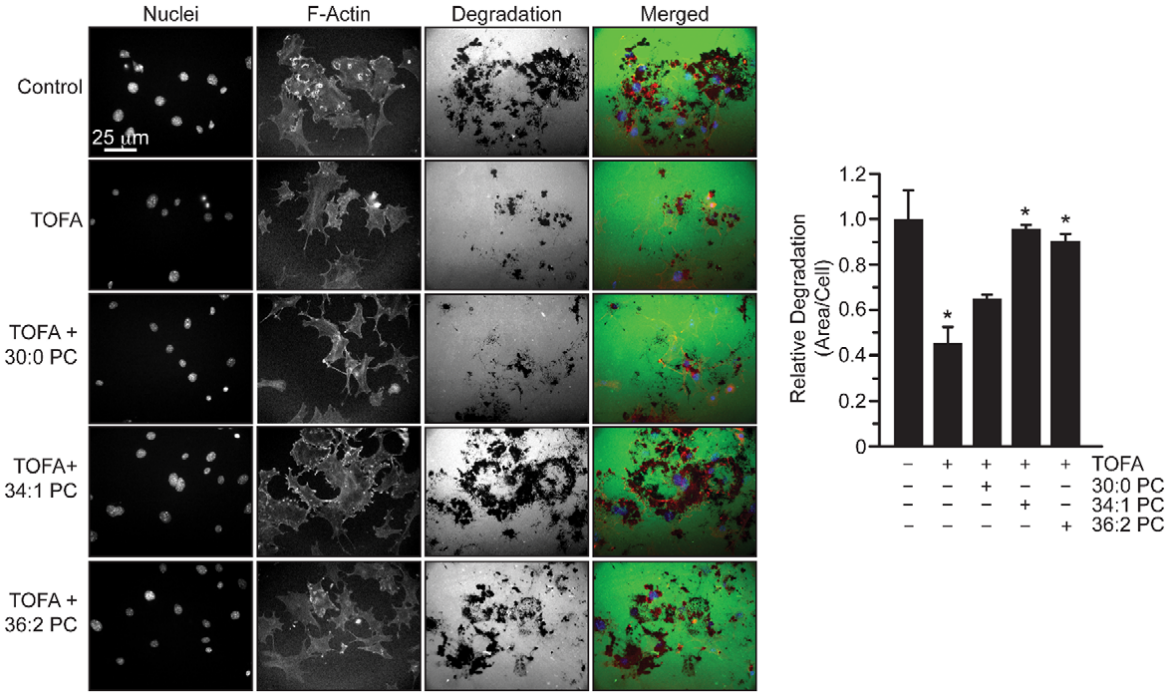
Phosphatidylcholine supplementation restores gelatin degradation in TOFA-treated 3T3-Src cells. Gelatin degradation of 3T3-Src cells seeded on glass cover slips coated with AlexaFluor-488 conjugated gelatin in the presence of vehicle, TOFA (30 µM), or TOFA with 30∶0 PC, 34∶1 PC, or 36∶2 PC for 24 hours. Nuclei were visualized by staining with DAPI and actin was visualized with Alexafluor594-phalloidin. Degraded area per cell is graphed. Scale bar, 25 µm. *p≤0.05.

Because invadopodia are linked with cellular invasion, we determined the effect of inhibiting ACC1 on the ability of 3T3-Src cells to degrade a gelatin matrix. Similar to the effect on invadopodia incidence (Fig. 1), TOFA treatment diminished the gelatin degrading activity of 3T3-Src cells by 60% (Fig. 5A, p≤0.001). The addition of 16∶0 and 18∶1 fatty acid restored the gelatin degrading capacity of TOFA-treated 3T3-Src cells to levels equal to or exceeding control (Fig. 5A, p≤0.05). Similarly, 48-hour knockdown of ACC1 decreased gelatin degradation by approximately 50%, which was completely restored by the addition of 18∶1 fatty acid (Fig. 5B, p≤0.05). Gelatin zymography was used to measure matrix metalloproteinase (MMP) activity in conditioned media from TOFA-treated 3T3-Src cells. Consistent with the effects on gelatin degradation *in situ*, TOFA treatment reduced the level of secreted MMP-2 that was restored by the addition of 18∶1 fatty acid (Fig. 5C). The ability of PC species to restore gelatin-degrading activity of TOFA-treated 3T3-Src cells was also investigated. Consistent with a lack of effect on invadopodia, the addition of 30∶0 PC to TOFA-treated 3T3-Src cells did not restore the gelatin degrading activity of the cells (Fig. 6). On the other hand, 34∶1 PC and 36∶2 PC restored the gelatin degrading activity of TOFA-treated 3T3-Src cells to approximately 95% of control (Fig. 6, p≤0.05).

### Inhibition of fatty acid synthesis by AMPK reduces invadopodia incidence

**Figure 7:**
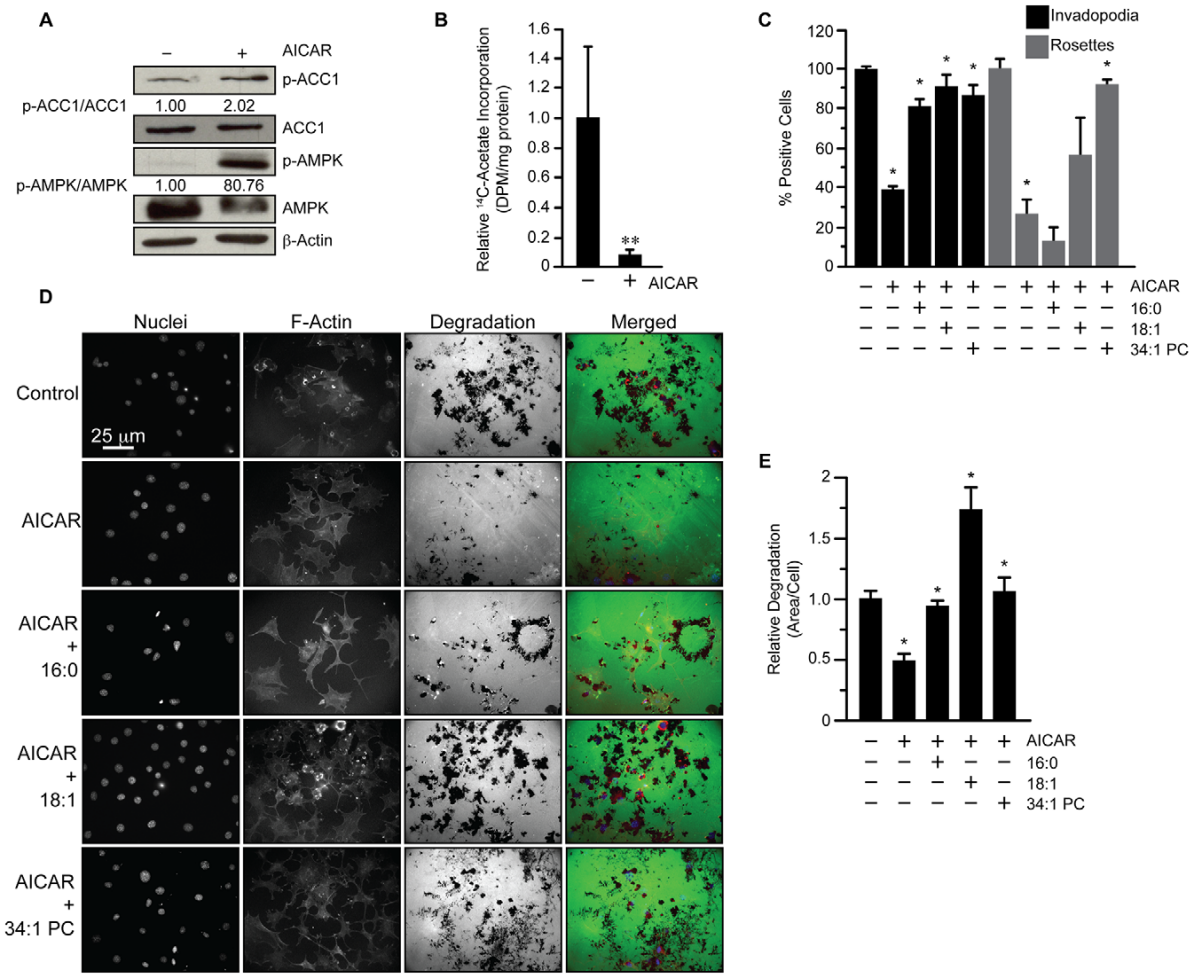
AMPK activation induces ACC1 phosphorylation and loss of invadopodia. (A) 3T3-Src cells were treated with AICAR (2.5 mM) for 16 hours and levels of p-ACC1, total ACC, p-AMPK, total AMPK, and β-actin were assessed by immunoblot. Band intensities were quantified using densitometry. (B) Fatty acid synthesis was determined by incorporation of ^14^C-acetate into lipid in 3T3-Src cells following treatment with AICAR (2.5 mM) for 16 hours. (C) Invadopodia incidence in 3T3-Src cells that were treated with vehicle or AICAR (2.5 mM) or AICAR + 16∶0 or 18∶1 fatty acid and 34∶1 PC 16 hours. Invadopodia and rosettes were quantified after AlexaFluor488-phalloidin staining. (D) Gelatin degradation of 3T3-Src cells seeded on AlexaFluor-488 conjugated gelatin coated glass coverslips in the presence of vehicle, AICAR (2.5 mM), or AICAR with 16∶0 or 18∶1 fatty acid and 34∶1 PC for 16 hours. Nuclei were visualized by staining with DAPI and actin was visualized with Alexafluor594-phalloidin. (E) Quantification of D. Scale bar, 25 µm. *p≤0.05.

We next determined whether inhibition of fatty acid synthesis through another mechanism could negatively impact invadopodia. Because ACC1 activity can be shut down via phosphorylation by the AMPK, the effect of AMPK activation on invadopodia formation and activity was assessed. Treatment with AICAR activated AMPK and induced a 2-fold increase in ACC1 phosphorylation (Fig. 7A), which corresponded with a 90% reduction in fatty acid synthesis (Fig. 7B, p≤0.05). Similar to the effects of ACC1 inhibition, AMPK activation and subsequent ACC1 phosphorylation reduced invadopodia incidence to 39% of control and rosette incidence to 27% of control (Fig. 7C, p≤0.05). The addition of 16∶0 or 18∶1 fatty acid and 34∶1 PC to AICAR-treated cells restored invadopodia presence to 81, 91, and 86% of control, respectively (Fig. 7C, p≤0.05). The addition of 34∶1 PC to AICAR-treated cells also restored rosette formation to 92% of control, whereas the addition of 16∶0 or 18∶1 fatty acid did not significantly rescue rosette incidence (Fig. 7C, p≤0.05). Consistent with the effect on invadopodia incidence, AICAR treatment also reduced gelatin degradation to 49% of control (Fig. 7D&E, p≤0.05). The addition of 16∶0 or 18∶1 fatty acid and 34∶1 PC to AICAR-treated cells restored gelatin degradation to 94, 172, and 106% of control levels, respectively (Fig. 7D&E, p≤0.05). Collectively, these data demonstrate that modulation of fatty acid synthesis through AMPK activation and ACC phosphorylation also impacts on invadopodia incidence and activity.

### ACC activity is required for cellular invasion

**Figure 8:**
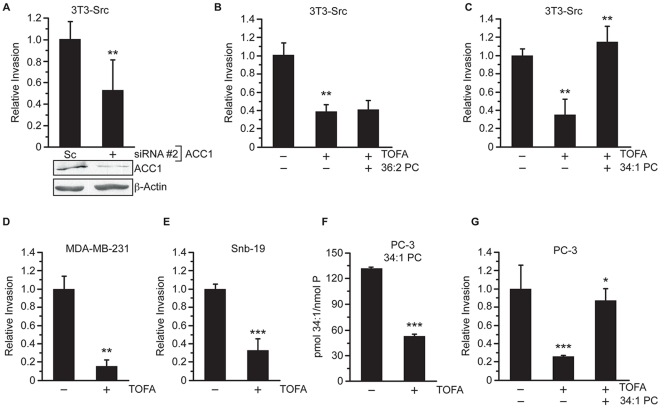
ACC activity is important for invasive capabilities and lipid composition of cancer cells. (A) Invasion through matrigel-coated invasion chambers was determined 3T3-Src cells transfected with 100 nM scrambled (Sc) or siRNA #2 against ACC1. Invasion was determined following staining with crystal violet. (B) Invasion through matrigel-coated invasion chambers was determined in 3T3-Src cells treated with vehicle, TOFA (30 µM), or TOFA + 36∶2 PC. Invasion was quantified as in A. (C) Invasion was determined in 3T3-Src cells treated with vehicle, TOFA (30 µM), or TOFA + 34∶1 PC. (D) MDA-MB-231 or (E) Snb-19 invasion through matrigel-coated chambers in the presence of vehicle or TOFA (30 µM). (F) The levels of 34∶1 PC in PC-3 cells treated vehicle or TOFA as determined by mass spectrometry. (G) Invasion of PC-3 cells through matrigel-coated invasion chambers in the presence of vehicle, TOFA (30 µM), or TOFA + 34∶1 PC. * p≤0.05; **p≤0.01; ***p≤0.001.

Because inhibition of ACC1 decreased invadopodia incidence and impaired the capacity of 3T3-Src cells to degrade gelatin, the impact on the invasive capacity of cells was also determined. Transfection of 3T3-Src cells with ACC1-specific siRNA reduced invasion through matrigel-coated membranes by 50% compared to cells transfected with scrambled siRNA (p≤0.01, Fig. 8A). Treatment of 3T3-Src cells with TOFA also reduced invasion through matrigel-coated membranes to 37% of control (Fig. 8B&C, p≤0.01). Interestingly, even though 36∶2 PC was able to fully restore invadopodia incidence in TOFA-treated cells, 36∶2 PC did not restore the ability of TOFA-treated 3T3-Src to invade through matrigel (p≤0.9; Fig. 8B). On the other hand, the addition of 34∶1 PC completely restored the invasive capacity of TOFA-treated 3T3-Src cells to control levels (p≤0.004, Fig. 8C). These data clearly demonstrate that fatty acid and lipid synthesis are required for cellular invasion, but that specific lipid species have defined roles in the invasive process.

The ability of TOFA to inhibit the invasive capacity of MDA-MB-231, Snb-19, and PC-3 human tumor cell lines, representing breast cancer, glioblastoma, and prostate cancer, respectively, was also determined. TOFA treatment reduced invasion of MDA-MB-231 and Snb19 cells through matrigel-coated membranes by 84% (p≤0.01) and 67% (p≤0.01), respectively (Fig. 8D&E). TOFA treatment also reduced the invasion of PC-3 cells through matrigel-coated membranes by 74% (p≤0.0001, Fig. 8G). The addition of 34∶1 PC, which was reduced by TOFA treatment (Fig. 8F), restored the invasive capacity of TOFA-treated PC-3 cells to 87% of control (p≤0.02, Fig. 8G). Combined with data demonstrating a role for ACC1 in invadopodia, these data suggest that ACC1 contributes to the lipid-dependent regulation of human tumor cell invasion.

## Discussion

Given the ubiquitous association of the fatty acid synthesis pathway in cancer, it is critical to clarify the roles the pathway plays in determining the behavior and function of tumor cells. Increased fatty acid synthesis has been noted in multiple cancers [18] and expression is associated with more aggressive disease [19], [20], [21], [22], [23], [24], [25], [26], [27], [28], [42]. Even though *de novo* fatty acid synthesis is clearly associated with membrane biogenesis, the mechanism by which the pathway regulates the processes of tumor cell invasion and migration has remained undefined. The data presented herein demonstrate that ACC1 is required for invadopodia formation and the invasive ability of tumor cells.

It has been demonstrated that in tumor cells most fatty acid that is synthesized *de novo* incorporates into glycerophospholipids and is used for membrane biogenesis [17]. Concomitant with this finding, it is also evident that much of the *de novo* synthesized fatty acid is enriched in lipid raft-like domains [17]. Conversely, inhibition of fatty acid synthesis appears to disrupt lipid raft domains. This is consistent with the notion that lipid rafts are primarily composed of phospholipids with saturated fatty acyl tails, along with cholesterol and sphingolipids [43]. This, combined with our finding that inhibition of fatty acid synthesis disrupts invadopodia (Fig. 1), suggests that fatty acid synthesis contributes to invadopodia by coordinating and maintaining lipid raft structures. Further support of this notion is the fact that lipid rafts are enriched at sites of invadopodia. Depletion of cholesterol levels reduces invadopodia incidence and gelatin degrading activity and reduces localization of the raft-associated protein caveolin-1 to invadopodia. [11], [12]. The transmembrane protease ADAM12, which also interacts with Tks5, is also known to localize to lipid rafts and caveolin 1 [44]. Taken together, these findings further connect lipid rafts and invadopodia.

Invadopodia are complex structures that require the coordinated interaction between the cellular membrane and the cytosolic invadopodia components [1]. Accordingly, several lipid species are intimately involved at sites of invadopodia nucleation, specifically, PI(3,4)-P_2_, PI(3,4,5)-P_3_, and PI(4,5)-P_2_ [8], [45]. Invadopodia initiate at focal adhesions where PI(3,4)P_2_ is enriched upon Src activation [8]. The activity of the p110α subunit of PI3 kinase is also necessary for invadopodia formation in breast cancer cells [9]. Several proteins encoding lipid-binding domains mediate the connection of invadopodia machinery to the cell membrane. For example, Tks4 and Tks5 bind to PI(3)-P and PI(3,4)-P_2_ through their PX domains [46], [47]. Moreover, the interaction of Tks5 with PI(3,4)P_2_ leads to a Tks5-Grb2 complex that is required for invadopodia formation [8]. In addition, N-WASp and dynamin bind PI(4,5)-P_2_ through their PH domains [48], [49], and gelsolin binds PI(3,4,5)-P_3_ through a non-classical PI binding domain which facilitates podosome initiation [50], [51]. Phosphoinositides can also cluster in lipid raft domains of the cellular membrane and cytoskeleton-membrane interactions can occur at sites of lipid rafts [52]. Although we did not profile phosphatidylinositol phosphates (PIPs) in this study, we did identify the overall change in the lipid profile associated with inhibition of fatty acid synthesis and loss of invadopodia. Importantly, the changes are consistent with what has been reported previously [40]. Moreover, the profiles allowed us to explore the role of specific lipid species in invadopodia.

This study focused on the contribution of fatty acids and PC lipids to invadopodia. PC is generally the most abundant class of glycerophopsholipids in cells and preferentially resides in the outer leaflet of the plasma membrane [41], [53]. Just as inhibition of fatty acid synthesis through ACC1 blockade reduces invadopodia, the addition of exogenous fatty acid to cells with depleted ACC1 activity restores them. It is interesting to note that in this system exogenous fatty acid was able to restore invadopodia to cells with blocked ACC1 activity, but rosettes were not always restored. Rosettes are generally considered superstructures of multiple invadopodia and are generally associated with Src-transformed cells. There is little information regarding a distinct role for each structure in the invasive process however. Regardless, our data clearly demonstrate that a full complement of rosettes is not required for the invasion of Src-transformed 3T3 cells. We further demonstrated that 34∶1 PC restores invadopodia (Fig. 4), restores the capacity to degrade gelatin (Fig. 6), and rescues the invasive capacity of tumor cells treated with ACC inhibitors or transfected with siRNA against ACC1 (Fig. 8). The 34∶1 PC species is the most abundant glycerophospholipid species in the 3T3-Src cells and includes 16∶0 and 18∶1 fatty acyl tails (Table 1). Because of this, we surmise that exogenous addition of 34∶1 PC populates the outer leaflet of the cellular membrane and restores the ability of cells to form lipid raft structures and to cluster PIPs appropriately to initiate invadopodia. It does not rule out the possibility that specific lipid-mediated signaling processes could also contribute to the formation of invadopodia. Interestingly, the 36∶2 lipid species was able to restore invadopodia and gelatin degradation (Figs. 4&6) but not invasion through matrigel (Fig. 8). These data suggest that even cells with a robust presence of invadopodia could be defective in the capacity to invade. Moreover, these findings support the idea that specific membrane composition and architecture is required for the processes that drive invadopodia and invasion. It will be interesting to identify whether invadopodia components differ in cells rescued with 36∶2 PC compared to 34∶1 PC. Our data further highlight the important role that lipids play in invadopodia, particularly PC lipids. The data do not, however, rule out that other lipids, PC species or other, having a role in invadopodia and the invasive process.

Inhibition of ACC disrupts the lipid composition of the cell membrane (Table 1). It reduces the overall level of saturated lipid and increases the relative level unsaturated species (Table 1). In doing so, inhibition of fatty acid synthesis may also affect the physical properties of membrane fluctuations. Interestingly, a body of literature has provided evidence to suggest that membrane dynamics are an important regulator of actin polymerization. In particular, membrane curvature in an important contributor to podosome and invadopodia assembly and is regulated by BAR domain containing proteins [13]. For example, Toca-1 forms positive membrane curvatures and is known to activate the N-WASp/Wip complex at sites of curvature [54], [55], [56], implying that invadopodia and podosome initiation requires proper membrane bending. It has also been shown that ASAP1 [57] and FBP17 [58] are critical players in podosome formation. Altered membrane phospholipid composition as a result of reduced ACC activity may impair the ability of BAR domain containing proteins to properly facilitate membrane bending and, by extension, the activation of critical invadopodia and podosome proteins.

There is a body of evidence that links AMPK activity to actin dynamics and polymerization. For example, pharmacological activation of AMPK induces astrocyte stellation characterized by F-actin disassembly and dispersion of focal adhesion complexes and also induces cytoskeletal rearrangement in epithelial and endothelial cells [59], [60], [61]. It has also been demonstrated that AMPK regulates secretory vesicles and virus entry through regulation of actin-dependent processes [62], [63]. Multiple effectors have been ascribed to AMPK in each process. Our data demonstrate that AMPK activation is associated with loss of invadopodia because of reduced F-actin polymerization (Fig. 7). Moreover, the effect is associated with reduced fatty acid synthesis, and invadopodia are rescued by the addition of fatty acid (Fig. 7). This is consistent with a role for fatty acid metabolism being required for membrane-cytoskeleton interactions. It also suggests that decreased fatty acid synthesis and changes in membrane lipid compositions could be the mechanism that links AMPK activity to the effects on actin cytoskeleton dynamics in other systems. Because AMPK has recently come to the forefront as a therapeutic target in cancer, it is worth exploring the ability of other AMPK activators to decrease invadopodia and the invasive phenotype.

Although invadopodia are frequently linked to the metastatic potential of tumor cells, a number of normal cells also utilize podosomes to facilitate degradation of extracellular components [1]. Osteoclasts are one type of normal cell that utilize podosomes for motility, adhesion and function [64]. Mice lacking the podosome protein Tks4 (SH3PXD2B) have bone abnormalities that result in skeletal dysplasia that is associated with Frank-Ter Haar Syndrome [65]. This suggests that loss of Tks4 could reduce podosome function in osteoclasts and negatively affect bone remodeling. Interestingly, mice with an aP2-Cre driven deletion of ACC1 in bone also display skeletal growth retardation and reduced osteoclast function [66]. Based on our observation that ACC1 inhibition reduces invadopodia incidence and activity, it is tempting to speculate that the bone phenotype associated with ACC1 deletion in mice is also due to decreased podosome activity.

Altogether, the combined data highlight the importance of ACC1 and *de novo* fatty acid synthesis in regulating invadopodia and the invasive properties of transformed cells. As mentioned above, ACC1 also contributes to fatty acid elongation. Our data suggest that its role in fatty acid synthesis is the predominant factor in invadopodia in that the exogenous addition of fatty acid restores the invadopodia phenotype to cells with blocked ACC activity. It appears that ACC may be necessary but not sufficient for invadopodia formation and the invasive capacity of tumor cells. Most cancer cells express ACC1 at relatively high levels, but only a subset of cancer cells make invadopodia. This would be akin to what has been described for PI3-kinase and invadopodia [9]. This study establishes a nexus between ACC1 activity, lipogenesis and invadopodia. In doing so, it also suggests that other lipid-regulated pathways may also be implicated in invadopodia and the invasive process. Because ACC1 is required for some of the processes associated with an invasive phenotype, it may be possible that pharmacological inhibition of ACC could reduce metastatic spread to distant sites as well as reducing localized tumor burden.
